# Risk factors of ovarian cancer: a systematic review and meta-analysis of Mendelian randomiation studies

**DOI:** 10.1093/pubmed/fdag034

**Published:** 2026-05-13

**Authors:** Melaku Yalew, Amanda L Lumsden, Anwar Mulugeta, Iqbal Madakkatel, Sang Hong Lee, Martin K Oehler, Johanna Mäenpää, Elina Hyppönen

**Affiliations:** Australian Centre for Precision Health, School of Public Health, Adelaide University, North Terrace, Adelaide CBD, SA 5000, Australia; South Australian Health and Medical Research Institute, North Terrace, Adelaide, SA 5000, Australia; Department of Public Health, College of Medicine and Health Sciences, Injibara University, Injibara, Ethiopia; Australian Centre for Precision Health, School of Public Health, Adelaide University, North Terrace, Adelaide CBD, SA 5000, Australia; South Australian Health and Medical Research Institute, North Terrace, Adelaide, SA 5000, Australia; Australian Centre for Precision Health, School of Public Health, Adelaide University, North Terrace, Adelaide CBD, SA 5000, Australia; South Australian Health and Medical Research Institute, North Terrace, Adelaide, SA 5000, Australia; Department of Pharmacology and Clinical Pharmacy, College of Health Sciences, Addis Ababa University, Addis Ababa, Ethiopia; Australian Centre for Precision Health, School of Public Health, Adelaide University, North Terrace, Adelaide CBD, SA 5000, Australia; South Australian Health and Medical Research Institute, North Terrace, Adelaide, SA 5000, Australia; Australian Centre for Precision Health, School of Public Health, Adelaide University, North Terrace, Adelaide CBD, SA 5000, Australia; South Australian Health and Medical Research Institute, North Terrace, Adelaide, SA 5000, Australia; Department of Gynaecological Oncology, Royal Adelaide Hospital, Port Road, Adelaide, SA 5000, Australia; Adelaide Medical School, Robinson Research Institute, Adelaide University, North Terrace, Adelaide, SA 5006, Australia; Faculty of Medicine and Medical Technology, Tampere University, Arvo Ylpön katu, 33014 Tampere, Finland; Cancer Centre, Tampere University and University Hospital, Elämänaukio, 33520 Tampere, Finland; Australian Centre for Precision Health, School of Public Health, Adelaide University, North Terrace, Adelaide CBD, SA 5000, Australia; South Australian Health and Medical Research Institute, North Terrace, Adelaide, SA 5000, Australia

**Keywords:** Ovarian cancer, Mendelian randomisation, systematic review, meta-analysis

## Abstract

**Background:**

Ovarian cancer (OC) remains a major global health issue, often diagnosed late and lacking effective screening.

**Methods:**

MR studies until 11 September 2023 were identified by a systematic search across nine databases. We complied with PRISMA guidelines and included different OC subtypes and all exposures studied, conducting meta-analyses where feasible to combine estimates from non-overlapping samples.

**Results:**

We identified 120 articles examining genetic evidence for an association between 230 exposures and OC risk. Endometriosis, late age at menopause, and several adiposity measures were robustly associated with greater OC risk. In contrast, late age at menarche, higher adiponectin, and body fat without adverse metabolic profile were associated with lower risk (favourable adiposity: meta-analysis OR per SD 0.35, 95% CI 0.20–0.61). Meta-analyses on lipid-lowering drug target HMG-CoA reductase inhibitor (OR 0.66, 95% CI 0.53–0.82), serum vitamin D (OR 0.88, 95% CI 0.82–0.95), and dried fruit intake (HR 0.61, 95% CI 0.41–0.91) were supportive of protective associations.

**Conclusions:**

Genetic evidence confirms OC risks associated with endometriosis, and age at menarche and menopause. While greater overall adiposity increases the risk, fat without an adverse metabolic profile appears protective. Associations between vitamin D and HMG-CoA reductase inhibition with OC risk warrant further study.

## Introduction

Ovarian cancer (OC) is the third most common and the deadliest gynaecological cancer.^1^^,^^2^ Epithelial ovarian cancer (EOC) accounts for over 90% of all cases, and it is further categorised into serous, endometrioid, clear cell, and mucinous OC subtypes.^3–5^ There is substantial evidence supporting the role of both genetic and environmental factors in the aetiology of OC,^4^^,^^6–8^ although the exact causes remain poorly understood and may vary by subtype. Much of the current evidence comes from observational studies; however, due to methodological limitations, drawing reliable causal inference is challenging.^9^ Mendelian randomisation (MR) offers a complementary genetic approach for assessing potential causality, using genetic variants as instrumental variables for the risk factor under investigation.^10^

A wide range of factors, including physical measures,^11–14^ diseases,^15–17^ lifestyles,^18–20^ biomarkers,^21–23^ and different micronutrients,^24^^,^^25^ have been examined for potential association with OC using the MR approach. While some studies suggest that factors such as BMI, coffee consumption, smoking, and vitamin D are associated with OC risk, other studies have failed to provide supportive evidence.^18^^,^^20^^,^^25^^,^^26^ Determining risk factors for OC is crucial for effective public health interventions.^27^^,^^28^ However, to date, no studies have conducted a systematic evaluation of all available MR evidence. A previous narrative review, which included 30 studies, reported promising evidence for a possible role of certain lifestyle factors, physical features, and biomarkers.^29^ In this exposure-wide systematic review, we examined 120 MR studies, substantially expanding the range of exposures investigated and increasing statistical power through *de novo* meta-analyses across independent study samples. Our aim was to establish and comprehensively synthetise evidence for potential predictors and actionable targets for OC prevention.

## Methods

### Literature search

The protocol was registered in the International Prospective Register of Systematic Reviews, registration number: CRD42023443912 and followed the Preferred Reporting Items for Systematic Review and Meta-Analysis guidelines.^30^^,^^31^ We conducted searches in Medline, Embase, Scopus, Web of Science, ScienceDirect, Cumulated Index to Nursing and Allied Health Literature, Cochrane, MedRxiv, and BioRxiv databases to identify relevant studies published before 11 September 2023, including preprints. The search strategy was implemented to identify studies of ‘OC’ AND ‘MR’, incorporating combinations of MeSH terms and appropriate Boolean operators (for the full list, see Supplementary Table S1).

### Study selection and data extraction

Eligible studies were MR analyses on OC regardless of exposure type. We excluded inaccessible full texts and irrelevant publication types (e.g. abstracts, commentaries). All retrieved studies were imported into Covidence,^32^ and duplicates were removed. Two reviewers (M.Y. and A.L.) independently screened titles/abstracts and reviewed full texts. Disagreements were resolved by a third reviewer (E.H.). Data were extracted into a Microsoft Excel spreadsheet by M.Y. and A.L., including author names, genetic instruments, MR estimates, confidence intervals (CIs), and other relevant details.

### Evaluation of evidence

Evidence robustness was assessed using criteria extended from Markozannes,^33^ considering pleiotropy, statistical significance (*P* < .05), and consistency across MR methods, with the classification process illustrated in Supplementary Fig. S1. Five categories were defined: robust, probable, suggestive, insufficient, and non-evaluable. Robust evidence required significant and concordant estimates across all MR methods without pleiotropy. If pleiotropy was present, only pleiotropy-robust methods were considered, and all had to show consistent, significant results. Probable evidence required a significant IVW result and concordant estimates across methods, or at least one significant pleiotropy-robust method with consistent estimates in the presence of pleiotropy. Suggestive evidence applied when one method showed significance with overlapping CI from others, regardless of pleiotropy. Insufficient evidence was assigned when estimates were nonsignificant or inconsistent, or when IVW was significant, but others were not, with non-overlapping CIs. Presence of pleiotropy was determined by findings from the MR-Egger intercept test and/or the MR-PRESSO Global test and could only be assessed when authors conducted related analyses. Non-evaluable evidence applied to studies lacking sensitivity analyses.

### Statistical analysis

For exposures assessed by multiple MR studies using the same outcome data source, we prioritized the study with the most cases. If more than one study used exactly the same data, we chose the study with the most genetic instruments indexing the exposure. We conducted de novo meta-analyses on 46 exposures with independent cohort data, focusing on overall OC. Random-effects models (DerSimonian–Laird) were used when heterogeneity (*I*^2^) exceeded 50%. Effect estimates were reported as in the original studies, with all but one reporting odds ratios (OR) with 95% CIs. In cases of pleiotropy, MR-Egger or MR-PRESSO estimates were used. All analyses were performed using STATA version 18. Further details on the methods have been provided in Supplementary Information 1.

## Results

### Study selection and characteristics

**Figure 1:**
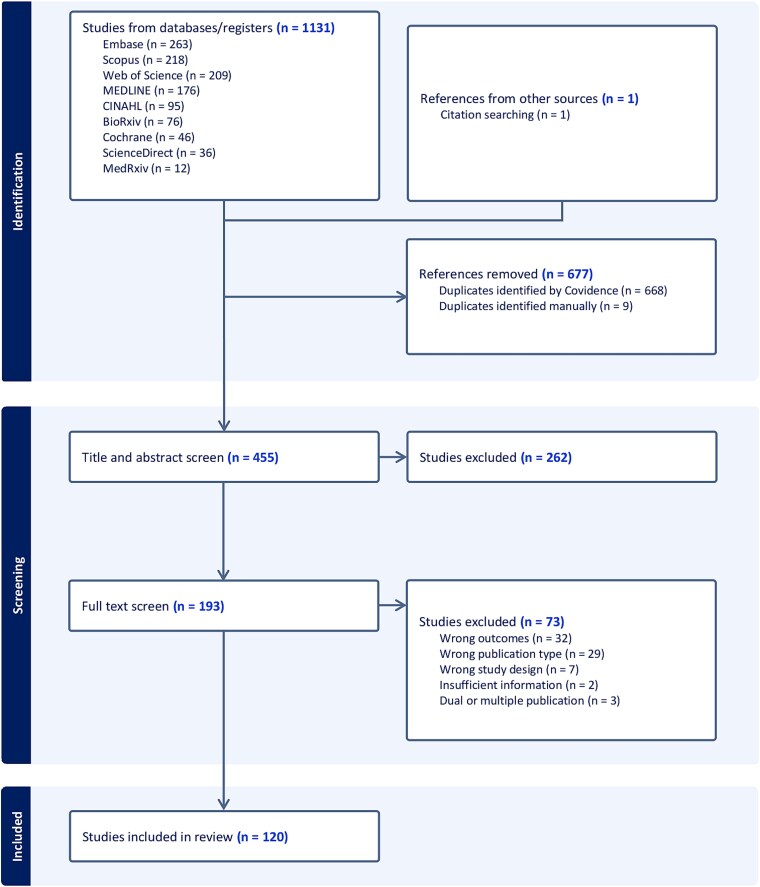
PRISMA flow chart showing the number of articles retrieved and excluded at each stage of the review.

From 1132 initially identified articles, 455 remained after removing 677 duplicates (Fig. 1). Title and abstract screening excluded 262, leaving 193 for full-text review. Of these, 120 met inclusion criteria, reporting 1964 associations across 230 genetically predicted exposures grouped into eight categories: physical measures (*n* = 27), reproductive factors (*n* = 7), lifestyle factors (*n* = 34), diseases/medical conditions (*n* = 32), drugs/drug targets (*n* = 10), nutrients (*n* = 29), biomarkers/metabolites (*n* = 72), and large screens (*n* = 19). Most studies (70.3%) used the Ovarian Cancer Association Consortium (OCAC, Phelan 2017)^34^ as the outcome source. See Supplementary Table S2 for exposure details and data sources.

### Robustness of evidence

Among the 1964 associations reported, 46 (2.34%) demonstrated robust evidence, while 105 (5.35%) indicated probable evidence. Of the total associations investigated, 805 (40.99%) were non-evaluable (13.25% of them were based on a single or two SNPs, when other MR methods could not be technically applied). Most of the robust associations were related to physical measures. A detailed breakdown of the evidence levels across the exposure categories is provided in Supplementary Table S3.

### Associations with physical measures

**Figure 2:**
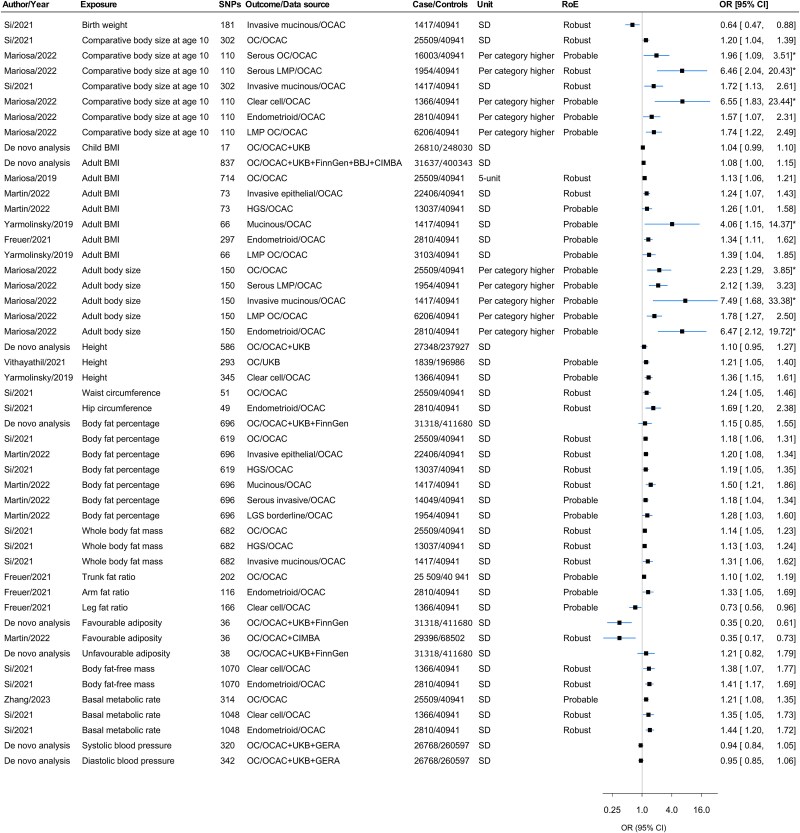
Forest plot for the associations of physical measures with OC and/or its subtypes. Abbreviations: BBJ, Bio-Bank Japan; BMI, body mass index; CIMBA, Consortium of Investigators for the Modifiers of BRCA1/2; GERA, Genetic Epidemiology Research on Adult Health and Aging; HGS, high-grade serous; LGS, low-grade serous; LMP, low malignant potential; NA, not available; OC, ovarian cancer; OCAC, Ovarian Cancer Association Consortium; OR, odds ratio; RoE, robustness of evidence; SD, standard deviation; UKB, UK Biobank; * = Estimates were taken from MR-Egger in the presence of horizontal pleiotropy.

Figure 2 shows MR evidence to support associations between physical measures and OC or its subtypes. Birth weight and comparative body size at age 10 years were robustly associated with invasive mucinous and overall OC, respectively.^20^^,^^35^ Adult BMI was consistently linked to a higher risk of overall and invasive OC,^20^^,^^35^^,^^36^ with meta-analysis suggesting 8% higher odds per SD (OR 1.08, 95% CI 1.002–1.15, *P*-value = .043) (Supplementary Fig. S2). Body fat percentage (BFP) was positively associated with overall and high-grade serous OC.^20^ Conversely, each SD increase in favourable adiposity (corresponding to 8.5% BFP)^36^ was associated with a 65% lower risk of OC (OR 0.35, 95% CI 0.20–0.61). Whole body fat mass was associated with overall OC, and various subtypes including high-grade serous OC^20^ (Fig. 2, Supplementary Table S4).

### Associations with reproductive factors

**Figure 3:**
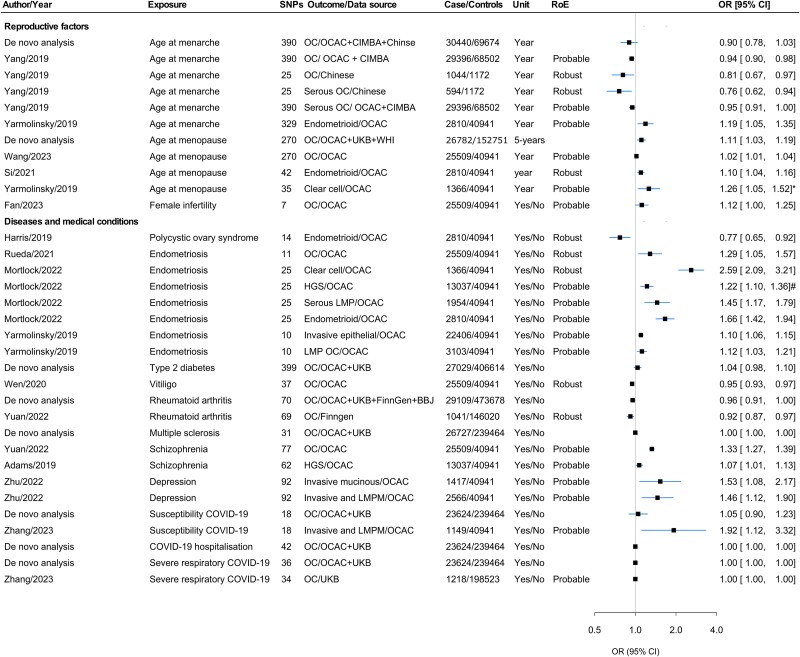
Forest plot for the associations of reproductive and disease- and medical condition–related factors with OC and/or its subtypes. Abbreviations: BBJ, Bio-Bank Japan; CIMBA, Consortium of Investigators for the Modifiers of BRCA1/2; HGS, high-grade serous; LMP, low malignant potential; LMPM, low malignant potential mucinous; NA, not available; OC, ovarian cancer; OCAC, Ovarian Cancer Association Consortium; OR, odds ratio; RoE, robustness of evidence; UKB, UK Biobank; WHI, Women Health Initiative; * = Estimates were taken from MR-Egger in the presence of horizontal pleiotropy; # = Estimates were taken from MR-PRESSO in the presence of horizontal pleiotropy.

Later age at menarche was associated with a lower risk of overall OC, as well as the serous subtype.^37–39^ Later age at natural menopause was associated with higher odds of OC (OR per 5 years 1.11, 95% CI 1.03–1.19), with a robust association seen for endometrioid OC.^20^^,^^38^ There was probable evidence for a borderline association between female infertility and a higher odds of OC^40^ (Fig. 3, Supplementary Table S5).

### Associations with diseases and medical conditions

Endometriosis showed robust links to overall and clear cell OC.^41^^,^^42^ Polycystic ovarian syndrome (PCOS) showed a robust inverse association with endometrioid OC.^39^^,^^43^ Among nonfemale-specific diseases, schizophrenia was positively associated with overall OC and high-grade serous OC.^44^ Both rheumatoid arthritis^45^ and vitiligo^46^ showed a robust negative association with OC. However, susceptibility to COVID-19 was linked with higher odds of low malignant mucinous OC in one study^47^ (Fig. 3, Supplementary Table S6).

### Association with lifestyle factors

**Figure 4:**
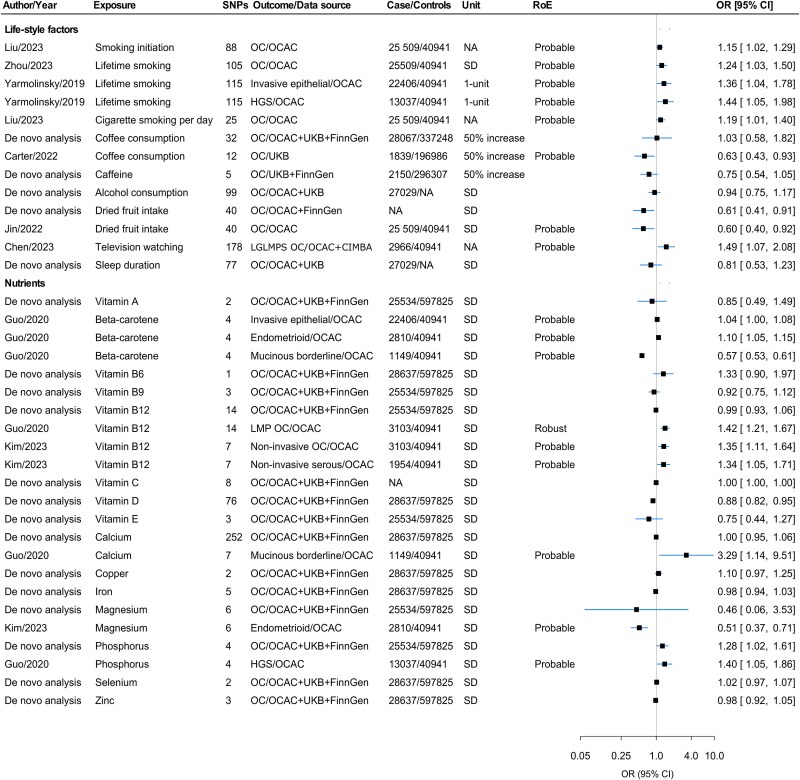
Forest plot for the associations of lifestyle- and nutrient-related factors with OC and/or its subtypes. Abbreviations: CIMBA, Consortium of Investigators for the Modifiers of BRCA1/2; HGS, high-grade serous OC; LGLMPS, low-grade and low malignant potential serous; LMP, low malignant potential; NA, not available; OC, ovarian cancer; OCAC, Ovarian Cancer Association Consortium; OR, odds ratio; RoE, robustness of evidence; SD, standard deviation; UKB, UK Biobank.

MR studies provided probable evidence for higher odds of OC associated with smoking history,^8^ and lifetime smoking index.^48^ From dietary factors, meta-analysis suggested a negative association between dried fruit intake and overall OC (HR per SD 0.61, 95% CI 0.41–0.91) (Fig. 4, Supplementary Table S7).

### Associations with circulating micronutrients

Vitamin B_12_ had a robust positive association with low malignant potential OC,^24^ while vitamin D concentrations (indexed by 25-hydroxyvitamin D) were negatively associated with overall OC (OR per SD 0.88, 95% CI 0.82–0.95). Magnesium showed a negative association with the endometrioid OC subtype,^49^ while phosphorus levels were positively associated with overall OC (OR per SD 1.28, 95% CI 1.02–1.61). Calcium levels were positively associated with mucinous borderline tumours^24^ (Fig. 4). Detailed descriptions of micronutrients associated with OC and/or its subtypes are provided in Supplementary Table S8.

### Associations with biomarkers and metabolites

**Figure 5:**
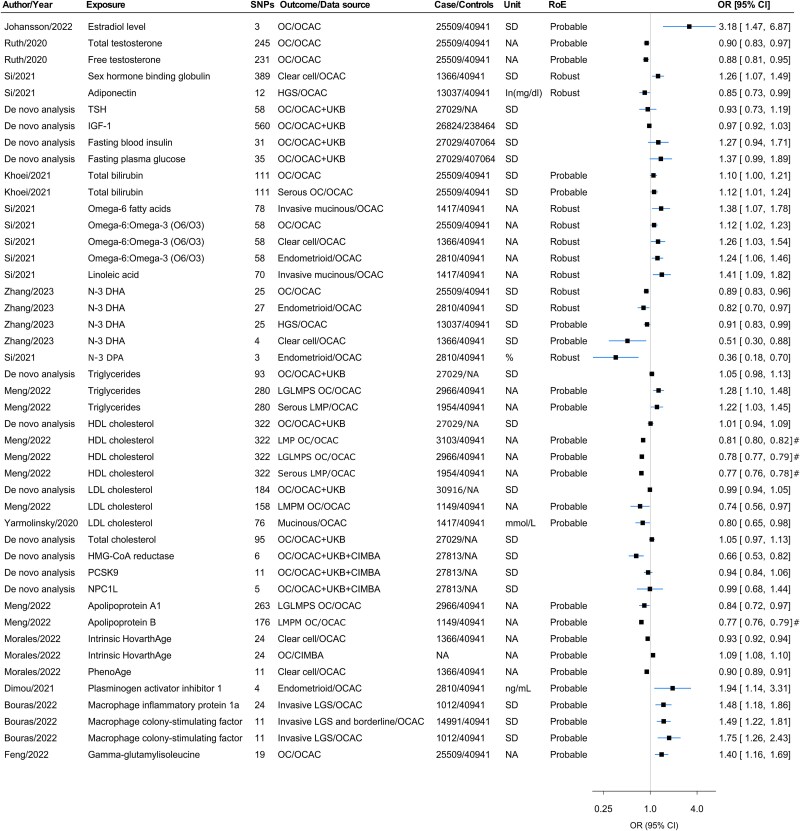
Forest plot for the associations of biomarkers with OC and/or its subtypes. Abbreviations: CIMBA, Consortium of Investigators for the Modifiers of BRCA1/2; DHA, docosahexaenoic acid; DPA, docosapentaenoic acid; HDL, high-density lipoprotein; HGS, high-grade serous; HMG-CoA, 3-hydroxy-3-methylglutaryl coenzyme A reductase inhibition; IGF-1, insulin-like growth factor 1; LGS, low-grade serous; LDL, low-density lipoprotein; LGLMPS, low-grade and low malignant potential serous; LMP, low malignant potential; LMPM, low malignant potential mucinous; NA, not available; NPC1L, Niemann–Pick C1-Like 1; OC, ovarian cancer; OCAC, Ovarian Cancer Association Consortium; OR, odds ratio; PCSK9, Proprotein convertase subtilisin/kexin type 9; RoE, robustness of evidence; SD, standard deviation; TSH, thyroid-stimulating hormone; UKB, UK Biobank; # = Estimates were taken from MR-PRESSO in the presence of horizontal pleiotropy.

Serum estradiol was positively associated with overall OC (OR per SD 3.18, 95% CI 1.47–6.87).^50^ Based on a screen by Si *et al*., consistent evidence across two MR approaches was reported for associations between sex hormone binding globulin (SHBG), adiponectin, omega-6 fatty acids, omega-6-to-omega-3 ratio (omega 6:3), linoleic acid, and N3 docosapentaenoic acid (N3-DPA) with overall OC and/or OC subtypes.^20^ There was probable evidence for an inverse association between high-density lipoprotein (HDL) cholesterol and low-malignant-potential OC subtypes.^51^ There were also probable associations between inflammatory biomarkers and some subtypes of OC (Fig. 5, Supplementary Table S9). Genetically indexed HMG-CoA reductase inhibition (a genetic proxy for statin use) was associated with a 34% lower odds of OC per SD increase (OR 0.66, 95% CI 0.53–0.82) (Fig. 5, Supplementary Table S10). Other evidence from studies on biomarkers, drug targets and large-scale screens was largely suggestive, insufficient, or non-evaluable,^22^^,^^48^^,^^52^^–^^58^ with details provided in Supplementary Tables S9–S11).

## Discussion

### Summary of main findings

This study synthesises genetic evidence on 230 OC risk factors sourced from 120 articles. MR studies confirm that hormone-related factors (such as age at menarche and menopause), disease conditions including endometriosis, and lifestyle and nutritional factors contribute to OC risk. The review highlights body composition and fat distribution as influential, with nuanced effects depending on metabolic impact. Some of the reported associations provide important promise for the possibility of reducing OC risk by modifiable factors.

### What is already known about this topic

Consistent with earlier studies,^59–65^ we found several measures indicative of higher adiposity, both during childhood and as an adult, to be genetically associated with OC risk. There are many plausible mechanisms that may underlie these associations, including adiposity-associated hormonal imbalance and inflammation.^66^^,^^67^ Adipokines secreted from the adipose tissue also contribute to angiogenesis,^68^^,^^69^ and obesity is a known risk factor for impaired immune function, which may reduce periodic clearance of cancer cells.^70^^,^^71^ It is interesting that favourable adiposity (indexed by genetic variants that characterise higher adiposity but low serum glucose, healthier lipid profiles, and low inflammation,^72^ was inversely associated with OC and also adiponectin), which is known for its anti-inflammatory properties,^73^^,^^74^ showed genetic evidence for a protective association. This potential association between adiponectin and OC is in line with previous observational studies.^68^ Notably, while triglycerides, linoleic acid, and the omega 6:3 ratio exhibited positive associations,^20^^,^^51^ docosahexaenoic acid (N3-DHA) and N3-DPA were negatively associated. Animal studies suggest that increased triglycerides and certain unsaturated fatty acids may be linked to insulin resistance and metabolic dysfunction, both of which are implicated in cancer development.^75^ However, the inverse association of DHA and DPA with OC might be due to cellular inhibition and apoptosis promotion.^76–78^ The genetic evidence for higher HDL cholesterol and HMG-CoA reductase inhibition with lower OC risk is supported by other evidence.^79^^,^^80^ In the MR setting, the HMG-CoA reductase inhibition (as there defined) can be taken to be equivalent for a genetic proxy equivalent to statin use, suggesting a potential to test for related effects in randomised experiments.

Older age at menopause and estradiol levels showed positive associations with OC, while late age at menarche and testosterone levels were inversely associated, consistent with prior studies.^63^^,^^81–83^ High or prolonged exposure to oestrogen over the reproductive years is implicated in increasing the risk of OC, possibly due to its role in promoting cell growth.^84^ However, the mechanism by which testosterone levels may contribute to OC risk reduction remains unclear, and further research is warranted. Endometriosis showed a positive association with OC, as frequently reported in previous studies,^63^^,^^85^ and might be linked to chronic inflammation or potential infertility.^86^ Furthermore, the endometrial tissue found outside the uterus is hormone-sensitive and may undergo frequent re-epithelisation during the reproductive period and ultimately lead to cancer. Hormonal influences might also explain many of the other associations, including the genetic evidence linking rheumatoid arthritis to OC. Indeed, premenopausal women with rheumatoid arthritis have been shown to have lower luteinizing hormone levels compared to healthy controls, which might affect ovulation and re-epithelisation.^87^ The observed protective association between PCOS and OC risk is plausibly explained by reduced ovulation frequency^88^ and higher testosterone levels.^89^ This MR evidence contradicts earlier observational studies that have reported increased^63^^,^^90^ or null risks,^91^^,^^92^ might reflect confounding or reverse association in non-genetic studies, and highlights the need for further study.

In line with previous findings,^93^^,^^94^ smoking was linked to an increased risk of OC, which may relate to carcinogenic substances in tobacco that promote DNA damage^95^^,^^96^ and provoke systemic inflammation and oxidative stress.^96^^,^^97^ Dried fruit intake was found to be inversely associated with OC, aligning with previous observational associations.^98^ A protective association might be explained by the high content of phytochemicals, carotenoids, and fibre in dried fruit.^99^^,^^100^ However, it’s uncertain whether genetic instruments can reliably capture such traits, and the role of population stratification and methodological biases requires further study. Vitamin D concentrations (indexed by 25-hydroxyvitamin D) were inversely associated with OC, consistent with previous findings.^101^^,^^102^ While the association deserves further study, the proposed anti-inflammatory and antioxidant effects,^103^ hormone-regulating properties, or the potential to induce apoptosis^104^ might mechanistically explain an association. We also observed evidence for OC-related links for other vitamins (notably, B12) and some micronutrients (notably calcium, magnesium and phosphorus) that may also warrant further study.

The positive association between schizophrenia and OC is consistent with findings from other studies.^105–107^ Although the underlying mechanisms remain unclear, patients with schizophrenia may have low parity, which could contribute to the association.^108^ Recent MR studies published after our main literature search have also identified OC related links with other diseases including uterine leiomyoma,^109^ hyperthyroidism,^110^ and ankylosing spondylitis,^111^^,^^112^ which may provide further insight into underlying mechanisms and possibly provide opportunities for disease monitoring. Increasing large-scale screening of blood and other biomarkers provides immense prospects for further discovery in this space. Research in this space is active, and while beyond the scope of our review, further insights have recently been gained on blood metabolites,^113–116^ gut microbiota,^115^^,^^117^^,^^118^ drug-target proteins,^119–121^ and DNA methylation.^122^ At the time of our review, 21 different metabolites and microbiota had been identified from large-scale screens of this kind; however, none of the associations were classified as robust.

### What this study adds

This study is the most comprehensive systematic review on OC risk factors and includes *de novo* meta-analyses of 46 risk factors. Evidence for the robustness of associations between OC and each exposure was evaluated using an adapted version of a prior approach that accounts for pleiotropy and the precision of estimation. While our study confirms the adverse role of excess adiposity, it also distinguishes favourable adiposity from general obesity, suggesting differential effects based on metabolic profile. Genetic evidence supports inverse associations with vitamin D and specific omega-3 fatty acids, alongside a positive association with triglycerides and other unsaturated fatty acids, while also strengthening evidence suggesting that genetically proxied inhibition of HMG-CoA reductase inhibition may play a protective role. Our study supports hormone-related influences on OC, makes links across disease outcomes, and provides new hope for modifiable metabolic and nutritional exposures in OC prevention.

### Limitations of the study

Most studies used genome-wide summary results from the OCAC to determine outcome associations; however, there was considerable heterogeneity across studies in both the number of genetic instruments used for exposure and the criteria to select those instruments. While most studies relied on genome-wide significance, 13% of the studies used only one or two SNPs, limiting the ability to assess pleiotropy. It is important to note that any evidence from MR studies will be only as valid as the instruments used to represent the exposure. Moreover, genetic instruments inherently capture uncertainty in the exposure measurement or definition, arising from the data or analyses in which they were identified. A further limitation reflects our inability to formally assess robustness of the evidence in a substantial proportion of studies, which were non-evaluable due to lack of sensitivity analyses. The evidence evaluation depended on the methodological approaches chosen by the authors and did not account for multiple testing. For some studies that considered multiple exposures, we identified issues with selective reporting where sensitivity analyses were presented for some, but not all, exposures investigated. Power to investigate associations was limited, especially for associations with OC subtypes, and even some robust evidence and pooled estimates were relatively imprecise with wide CIs. As our search included preprint repositories, two of the included studies were not peer-reviewed, while many of the studies identified were based on overlapping samples, which limited our ability to conduct meta-analyses. Formal assessment of publication bias was not performed because the number of studies per meta-analysis was small. Finally, the included studies will reflect methodological limitations inherent to MR, and none accounted for potential heterogeneity that may arise from gene–environment interaction and non-linear exposure associations. Consistent with current data availability, >95% of the studies included in this review were conducted in European populations, which may limit the generalizability to other ancestry groups.

## Conclusions

Genetic evidence supports established links between OC risk and factors including endometriosis, age at menarche and menopause. While greater overall adiposity appears to increase the risk of OC in MR studies, metabolically favourable adiposity had a protective association, indicating that the effects of adiposity on OC risk may depend on metabolic profile. Importantly, the findings highlight modifiable risk factors such as vitamin D, omega-3 fatty acids, and HMG-CoA reductase inhibition as having potential relevance for OC risk, meriting further research.

## Supplementary Material

Supplementary_information_1_fdag034

Supplementary_Tables_fdag034

## Data Availability

The data used/analysed in this study are attached with the manuscript.

## Notes

**Melaku Yalew**, Mr.

**Amanda L. Lumsden**, Dr.

**Anwar Mulugeta**, Dr.

**Iqbal Madakkatel**, Dr.

**S. Hong Lee**, Associate professor

**Martin K. Oehler**, Professor

**Johanna Mäenpää**, Professor

**Elina Hyppönen**, Professor
