# Supplementary material for: Risk factors of ovarian cancer: a systematic review and meta-analysis of Mendelian randomiation studies
Source: J Public Health (Oxf). 2026 May 13;48(2):430–43. doi: 10.1093/pubmed/fdag034 (PMC13223590; doi:10.1093/pubmed/fdag034)
Supplement: Supplementary_information_1_fdag034 [file supplementary_information_1_fdag034.docx]

**Supplementary information**

**Risk factors of ovarian cancer: A systematic review and meta-analysis of Mendelian randomisation studies**

Melaku Yalew ^1,2,3^, Amanda L. Lumsden ^1,2^, Anwar Mulugeta ^1,2,4^, Iqbal Madakkatel ^1,2^, S. Hong Lee ^1,2^, Martin K. Oehler ^5,6^, Johanna Mäenpää ^7,8^, Elina Hyppönen ^1,2*^

^1^Australian Centre for Precision Health, School of Public Health, Adelaide University, Adelaide 5001, Australia

^2^South Australian Health and Medical Research Institute, Adelaide 5000, Australia

^3^Department of Public Health, College of Medicine and Health Sciences, Injibara University, Injibara P.O. Box 6040, Ethiopia

^4^Department of Pharmacology and Clinical Pharmacy, College of Health Sciences, Addis Ababa University, Addis Ababa P.O. Box 9086, Ethiopia

^5^Department of Gynaecological Oncology, Royal Adelaide Hospital, Adelaide, SA 5000, Australia

^6^Adelaide Medical School, Robinson Research Institute, Adelaide University, Adelaide, SA 5006, Australia

^7^Faculty of Medicine and Medical Technology, Tampere University, 33014 Tampere, Finland

^8^Cancer Centre, Tampere University and University Hospital, 33520 Tampere, Finland

***Correspondence:** Professor Elina Hyppönen: Australian Centre for Precision Health, School of Public Health, Adelaide University, SAHMRI, Adelaide 5001, Australia

**Email address:** [**elina.hypponen@adelaide.edu.au**](mailto:elina.hypponen@adelaide.edu.au)

**Contents**

| Table | Title |
| --- | --- |
| ST 1 | Supplementary Table 1: Concepts, key/MeSH terms, search strategies and retrieved articles in each terms and combinations used in each database. |
| ST 2 | Supplementary Table 2: Summary of exposures and data sources used to assess genetic evidence supporting a causal association with ovarian cancer in MR studies. |
| ST 3 | Supplementary Table 3. Classification of associations between exposures, and OC and its subtypes, by levels of evidence. |
| ST 4 | Supplementary Table 4: Physical measure and related factors and their association with OC and/or the subtypes. |
| ST 5 | Supplementary Table 5: Reproductive and related factors and their association with OC and/or the subtypes. |
| ST 6 | Supplementary Table 6: Association with diseases, medical conditions and related factors and their association with OC and/or the subtypes. |
| ST 7 | Supplementary Table 7: Lifestyle and related factors and their association with OC and/or the subtypes. |
| ST 8 | Supplementary Table 8: Nutrients and their association with OC and/or the subtypes. |
| ST 9 | Supplementary Table 9: Biomarkers and related factors and their association with OC and/or the subtypes. |
| ST 10 | Supplementary Table 10: Drug and drug targets and their association with OC and/or the subtypes. |
| ST 11 | Supplementary Table 11: Large screens and their association with OC and/or the subtypes. |

**Supplementary methods**

The protocol was registered in the International Prospective Register of Systematic Reviews (PROSPERO), registration number: CRD42023443912. The systematic review was done in accordance with the Preferred Reporting Items for Systematic Review and Meta-Analysis (PRISMA) guidelines ^1, 2^.

**Literature search**

We conducted searches in Medline, Embase, Scopus, Web of Science, ScienceDirect, Cumulated Index to Nursing and Allied Health Literature (CINAHL), Cochrane, MedRxiv, and BioRxiv databases including all studies published before September 11, 2023. Articles, including pre-prints, were retrieved using a pre-specified search criteria using a combination of terms to capture the outcome. We included relevant MeSH terms and combined the search terms with appropriate Boolean operators as required. For the full search strategy, see **Supplementary Table 1**.

We included all MR studies conducted on adults on OC or its subtypes, with no restrictions on the exposure investigated. Full-length studies that were unavailable after contacting the corresponding author, or that were of the wrong publication type (such as conference or poster abstracts, commentaries, study protocols and reviews) were excluded. Additionally, publications that were not MR studies, were not on OC, or that provided insufficient information were not considered. For duplicate studies, we included the most recent version, with preference for peer-reviewed journal versions over preprints.

**Study selection and data extraction**

All studies retrieved from the databases were imported into Covidence software, and duplicate records were removed ^3^. A two-stage study selection process was conducted, consisting of preliminary and final stages. During the initial selection, two independent reviewers (M.Y. and A.L.) performed title and abstract screening for all the articles using predetermined inclusion and exclusion criteria, followed by full text review for the remaining articles. Any disagreement between the two reviewers were resolved by a third reviewer (E.H.). Data extraction was performed using a Microsoft Excel spreadsheet by two reviewers (M.Y. and A.L.). Information extracted included authors’ name, sample size, study population, ancestry, details of the genetic instrument, variance of exposure explained by the genetic instrument, MR effect estimates, 95% CIs and P-values (main and sensitivity analyses), and other relevant details.

**Evaluation of evidence**

**Supplementary Figure 1** provides illustration for the assessment of robustness of evidence. Our assessment accounted for the presence of pleiotropy, in addition to statistical significance (P<0.05) and concordance of effect estimates across MR methods, extended from a previously published criteria ^4^. Evidence was categorised into five levels: robust, probable, suggestive, insufficient, and non-evaluable, reflecting the strength of causal evidence rather than study quality. ***Robust evidence*** required statistically significant, concordant effect estimates across all MR methods in the absence of pleiotropy. If pleiotropy was present, only pleiotropy-robust methods were considered, with all expected to provide consistent and significant estimates. ***Probable evidence*** was assigned when, in the absence of pleiotropy, the primary analysis (IVW) was significant, and all MR methods showed directionally concordant estimates. With pleiotropy, at least one pleiotropy-robust method had to be significant, with concordant estimates across pleiotropy-robust methods. ***Suggestive evidence*** applied when, without pleiotropy, at least one MR method showed a significant association, with overlapping confidence intervals from other methods. When pleiotropy was present, at least one pleiotropy-robust method had to indicate a significant association with overlapping confidence intervals. ***Insufficient evidence*** was assigned when MR methods produced nonsignificant or directionally inconsistent estimates. In the absence of pleiotropy, evidence was also deemed insufficient if the primary analysis (IVW) was significant but other methods were not, with at least one showing a non-overlapping confidence interval. ***Non-evaluable evidence*** was assigned to studies lacking sensitivity analyses. This included two-sample MR studies that did not conduct or report sensitivity analyses, studies using only one or two genetic variants where sensitivity analyses are not technically applicable, as well as GRS-based MR studies or one-sample MR analyses relying on a single instrument. **Supplementary Figure 1** provides a detailed flowchart illustrating how the association falls at each level of evidence. The results description and conclusions, were made by prioritise to robust and probable associations, giving precedence to robust associations when both types are reported for the same exposure-outcome association in different studies.

**Statistical analysis**

When multiple MR studies investigated the same exposures using the same outcome data source (e.g., earlier versus later versions of consortia), we prioritized studies with the largest number of cases. For the selected outcome data source, in instances where more than two studies examined the same exposures, we selected the study with the largest number of genetic instruments. We conducted “de novo” meta-analyses on 46 exposures for which data were available on independent population cohorts for the outcome, focusing solely on overall OC. In instances where the reported exposure-outcome association exhibited heterogeneity between studies (I^2^ % greater than 50), a random-effects model was used. MR effect estimates in the meta-analyses reported as odds ratios (OR) with 95% confidence intervals (CI), except for dried fruit intake, which was presented as a hazard ratio (HR). In the presence of pleiotropy, estimates from pleiotropy-robust methods (MR-Egger or MR-PRESSO) were used. Where the identified papers included meta-analyses of the MR estimates from different populations and there were no further studies to include, we present the findings as reported in the original publication. All statistical analyses were performed using STATA version 18.

**Supplementary results**

**Study selection and characteristics**

The initial search identified 1,132 articles. After excluding 677 duplicates, the remaining 455 article underwent further screening (**Figure 1**). Based on titles and abstracts, we excluded 262 articles leaving 193 articles to full-text review. Of these 73 were subsequently excluded, while 120 articles met the inclusion criteria and were deemed eligible. These articles reported 1964 associations with OC or its subtypes, reflecting 230 separate genetically predicted exposures. We categorised the exposures into eight broad groups including physical measures (n=27), reproductive factors (n=7), lifestyle factors (n=34), diseases and medical conditions (n=32), drugs and drug targets (n=10), nutrients (n=29), biomarkers and metabolites (n=72), and large screens (n=19). Two-sample MR analyses were conducted in 94.2% of the included studies, while 2.5% employed both one-sample and two-sample methods. Multivariable MR analyses were considered for 17 exposures. The total sample size used to define genetic variant–exposure associations varied from 441 to 806,834, with the number of genetic variants instrumenting the exposure ranging from 1 to 2059 SNPs (inclusion threshold P<1×10^-11^ to P<1×10^-4^). One hundred ten (47.83%) of the exposures used a threshold below GWAS significant. The sample size to determine genetic variant–outcome associations varied between 1,740 and 560,012, with the number of OC cases ranging from 134 to 30,869. Many studies (70.3%) used the Ovarian Cancer Association Consortium (OCAC, Phelan 2017) as the primary outcome data source. Most studies looked at overall epithelial OC, with studies also conducted on serous, endometrioid, clear cell, and mucinous subtypes. A detailed description of all factors investigated in this review, along with the data sources, can be found in **Supplementary Table 2**.

**Robustness of evidence**

Each exposure to OC/subtype association was categorised into one of the five levels of evidence outlined in the methods section. Among the 1,964 associations reported, 46 (2.34%) demonstrated robust evidence, while 105 (5.35%) indicated probable evidence. Of the total associations investigated, 805 (40.99%) were non-evaluable, typically representing results from studies where sensitivity analyses had not been conducted (13.25% were based on a single or two SNPs, where other MR method options could not be technically applied). Most of the high-evidence associations were related to physical measures, followed by biomarkers. A detailed breakdown of the evidence levels across exposure categories is provided in **Supplementary Table 3.**

**Associations with physical measures**

As shown in **Figure 2**, MR studies provided genetic evidence to support associations between several physical measures and OC or its subtypes. Birth weight and comparative body size at age 10 years were robustly associated with invasive mucinous and overall OC, respectively ^5, 6^. Comparative body size at age 10 years was also robustly associated with low malignant potential serous and invasive mucinous OC. Adult BMI was consistently linked to higher risk of overall and invasive OC ^5-7^, with our meta-analysis supporting 8% higher odds of OC per SD higher (OR 1.08, 95% CI 1.00-1.15) (**Supplementary Figure 2**). This positive association extends to probable level for specific OC subtypes, including high-grade serous ^7^, endometrioid ^8, 9^, and mucinous ^9^. There was a robust positive association between waist circumference (WC) ^5^ and OC, and hip circumference (HC) and the endometrioid subtype ^5^. Overall body fat percentage (BFP) was positively associated with overall, and high-grade serous OC ^5^. Conversely, for favourable adiposity, defined as higher body fat percentage without metabolic abnormalities ^10, 11^, our meta-analysis supported a protective association with overall OC (OR per SD 0.35, 95% CI 0.20-0.61) (**Figure 2**).

There was genetic evidence for a positive association between whole body fat mass and overall OC, and various subtypes including high-grade serous, and invasive mucinous ^5^ **(Figure 2)**. In a study investigating the effects of body fat distribution on female cancers, trunk fat ratio (TFR; the ratio of fat mass in the trunk to total body fat mass) showed a probable positive association with overall OC ^8^. This association also supported in multivariate MR (controlling for arm fat ratio) for OC, endometroid, and clear cell subtypes ^8^. Both body fat-free mass and basal metabolic rate (BMR) had robust positive associations with clear cell and endometrioid OC subtypes in OCAC ^5^ (**Figure 2**). Taller height showed a probable association with OC and the clear cell subtype ^12^. A detailed description of all the physical measures investigated is included in **Supplementary Table 4**.

**Associations with reproductive factors**

As shown in **Figure 3**, various reproductive factors were tested for their causal association with OC. There was a robust negative association between age at menarche and overall OC, as well as with the serous subtype, in a Chinese population ^13^. There was probable evidence supporting inverse genetic associations between age at menarche and OC and certain subtypes ^13-15^ in populations of European ancestry, which was also shown in multivariate MR controlled for BMI and educational attainment ^14^ (**Supplementary Table 5**). A meta-analysis by Yang et al., encompassing *BRCA* mutation carriers and the general population, reported a probable inverse association between age at menarche and serous OC ^13^.

Later age at natural menopause was associated with higher odds of OC in univariate MR analyses (OR per five years 1.11, 95% CI 1.03-1.19) (**Figure 3, Supplementary Figure 2**), with a robust association seen for endometrioid OC ^5^ (**Figure 3**). There was also probable evidence supporting a negative association between later age at menopause and overall OC, shown by univariate analysis as well as in a multivariate MR analysis controlling for BMI and educational attainment ^14^. There was probable evidence for a borderline association between female infertility and a higher odds of OC ^16^ (**Figure 3, Supplementary Table 5**).

**Associations with diseases and medical conditions**

Various diseases and clinical conditions have been identified as having causal links with both overall OC and its subtypes ^17-20^ (**Figure 3**). There was robust evidence for an association between endometriosis and overall OC ^21^, as well as with the clear cell subtype ^22^ (**Figure 3, and Supplementary Table 6**). Probable evidence was observed for associations between endometriosis and several other subtypes: high-grade serous, endometrioid, invasive epithelial, low malignant serous and low malignant potential OC ^9, 22^. In contrast, a robust negative association has been documented between polycystic ovarian syndrome (PCOS) and endometrioid OC ^9, 23^ (**Figure 3**).

From non-female specific diseases, schizophrenia has shown probable positive associations with overall OC, and high-grade serous OC ^24^. There was also some borderline evidence for an inverse association between rheumatoid arthritis and overall OC (de novo meta-analysis). However, this association was no longer observed after removing estimates influenced by pleiotropy. In the FinnGen population, a robust negative association was observed between rheumatoid arthritis and OC ^17^. Vitiligo was another disease showing a robust inverse association with overall OC ^19^. However, susceptibility to COVID-19 have been reported with higher odds of low malignant mucinous OC ^25^ (**Supplementary Table 6**).

**Association with lifestyle factors**

MR studies provided probable evidence for higher odds of OC associated with smoking history ^26^, and lifetime smoking index ^27^ with a 15 % and 24% increase in OC risk per SD, respectively (**Figure 4**). Probable evidence supported similar associations for high-grade serous and invasive epithelial OC ^9^. A study on coffee consumption showed a probable negative association with OC in the UKB ^28^, however, our meta-analysis extending the data to OCAC and FinnGen found no evidence of an association with overall OC (OR per 50% increase in coffee consumption 1.03, 95% CI 0.58-1.82). Several studies examined associations between physical activity and OC or its subtypes. Suggestive evidence was observed for associations between overall physical activity and accelerator-based physical activity and endometrioid and mucinous OC subtypes ^29^. However, the evidence was insufficient for overall OC (OR 1.11, 95% CI 0.66-1.85) (**Supplementary Table 7**) ^30^. Probable evidence was reported supporting an association between television watching and higher risk of low-grade low malignant potential serous OC ^31^. From dietary factors, our meta-analysis suggested a negative association between dried fruit intake and overall OC (HR per SD 0.61, 95% CI 0.41-0.91) (**Figure 4**), while no conclusive evidence was found to support an association by other dietary factors, such as protein, carbohydrate, fat, or dietary vitamin E intake (**Supplementary Table 7**).

**Associations with circulating micronutrients**

Beta-carotene showed varied probable associations with different OC subtypes. It was positively associated with invasive OC and endometrioid OC, but negatively with mucinous borderline tumours ^32^. Meta-analysis did not provide evidence for an association between retinol (Vitamin A) and OC (**Figure 4, Supplementary Figure 2**). Vitamin B_12_ demonstrated a robust positive association with low malignant potential OC ^32^, and probable positive associations with non-invasive OC, non-invasive serous, and clear cell OC ^33^, however, we did not find any links for vitamin B_6_, B_9_ and B_12_ in our meta-analyses. Our meta-analysis showed that vitamin D was negatively associated with overall OC (OR per SD 0.88, 95% CI 0.82-0.95).

Magnesium showed a negative association with the endometrioid OC subtype with probable evidence ^33^, however, a meta-analysis on overall OC found no association (OR per SD 0.46, 95% CI 0.06-3.53). While individual studies showed a probable positive association between phosphorus and the high-grade serous subtype only ^32^, our meta-analysis suggested a positive association with overall OC (OR per SD 1.28, 95% CI 1.02-1.61). Calcium was positively associated with mucinous borderline tumours with probable evidence ^32^, however, our meta-analysis did not support an association with overall OC. Detailed descriptions of micronutrients (vitamins and minerals) associated with OC and/or its subtypes are provided in **Supplementary Table 8**.

**Associations with biomarkers and metabolites**

Serum estradiol was positively associated with overall OC (OR per SD 3.18, 95% CI 1.47-6.87) with probable evidence ^34^. For several biomarkers the largest study identified was the screen by Si et al. ^5^ which covered 55 biomarkers (among other exposures). Based on consistent evidence across two MR approaches implemented they reported evidence for associations for sex hormone binding globulin (SHBG), serum adiponectin, omega-6 fatty acids, omega-6 to omega-3 ratio, linoleic acid, and N3 Docosapentaenoic Acid (N3-DPA) with overall OC and/or OC subtypes (**Figure 5, Supplementary Table 9**). Of other lipid metabolites, there was probable evidence for an inverse association between high density lipoprotein (HDL) cholesterol and low malignant potential OC subtypes ^35^, while low density lipoprotein (LDL) cholestrol was negatively associated with mucinous OC ^36^ and low malignant potential mucinous OC ^35^. However, in our meta-analysis using data from OCAC and UKB, LDL and HDL were not associated with overall OC (**Figure 5, Supplementary Figure 2**). Genetically instrumented lipid lowering-based MR targeting PCSK9 and NPC1L1 was not associated with OC risk, while lipid lowering targeting HMG-CoA reductase inhibition was associated with a 34% lower odds of OC per SD increase (OR 0.66, 95% CI 0.53-0.82) (**Figure 5, Supplementary Table 10**).

Apolipoprotein A1 and apolipoprotein B showed probable negative associations with low-grade low malignant serous and low malignant mucinous OC, respectively ^35^. There were also some probable associations observed between inflammatory biomarkers and some subtypes of OC (**Figure 5, Supplementary Table 9**). From each category of exposure, various other associations were either deemed suggestive, had insufficient evidence, or were non-evaluable ^27, 36-42^ (**Supplementary Table 4 to 11**).

**Supplementary figures**


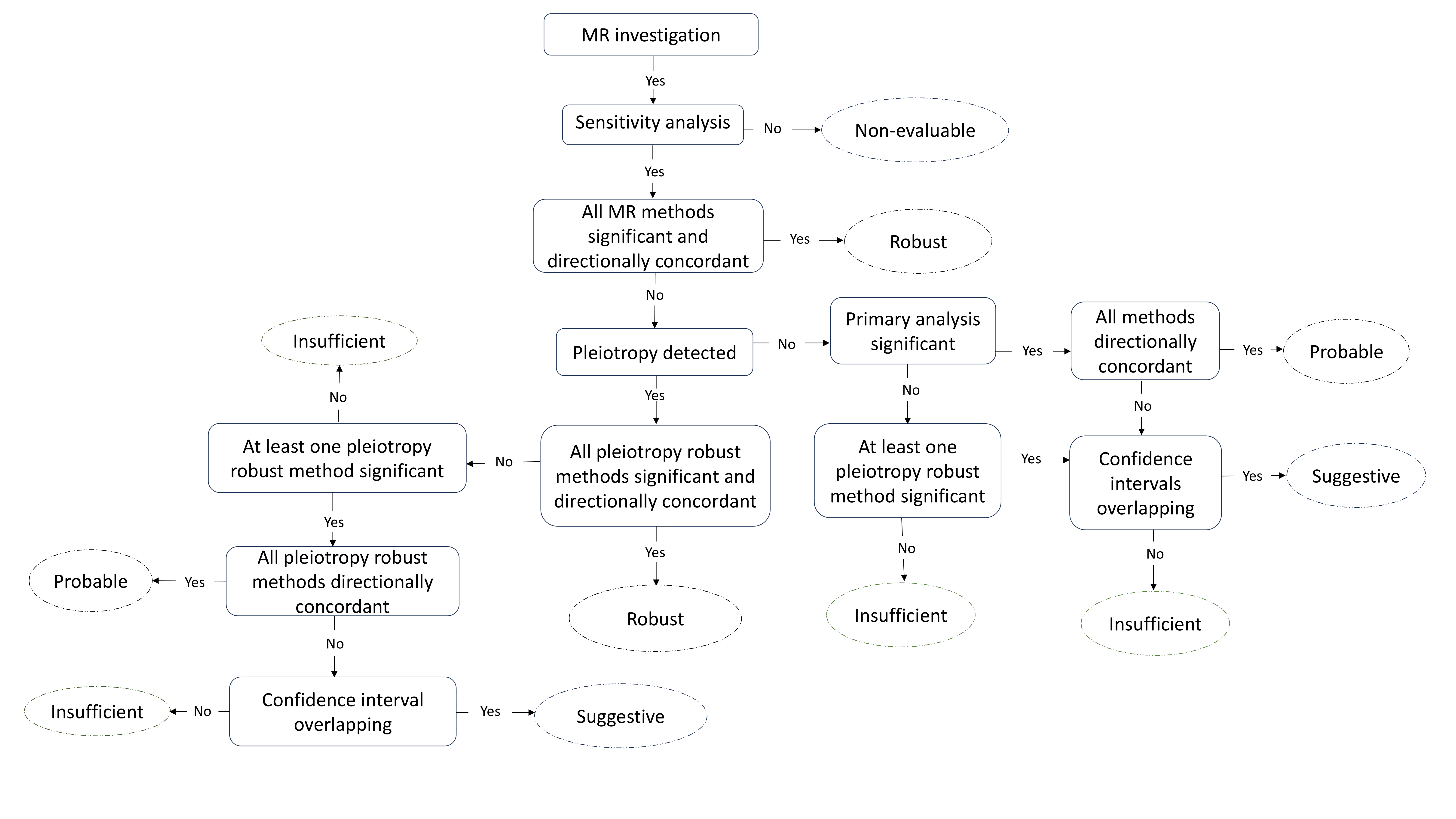
Supplementary Figure 1: Revised Markozannes robustness of evaluation flow chart showing how the associations categorised.

**
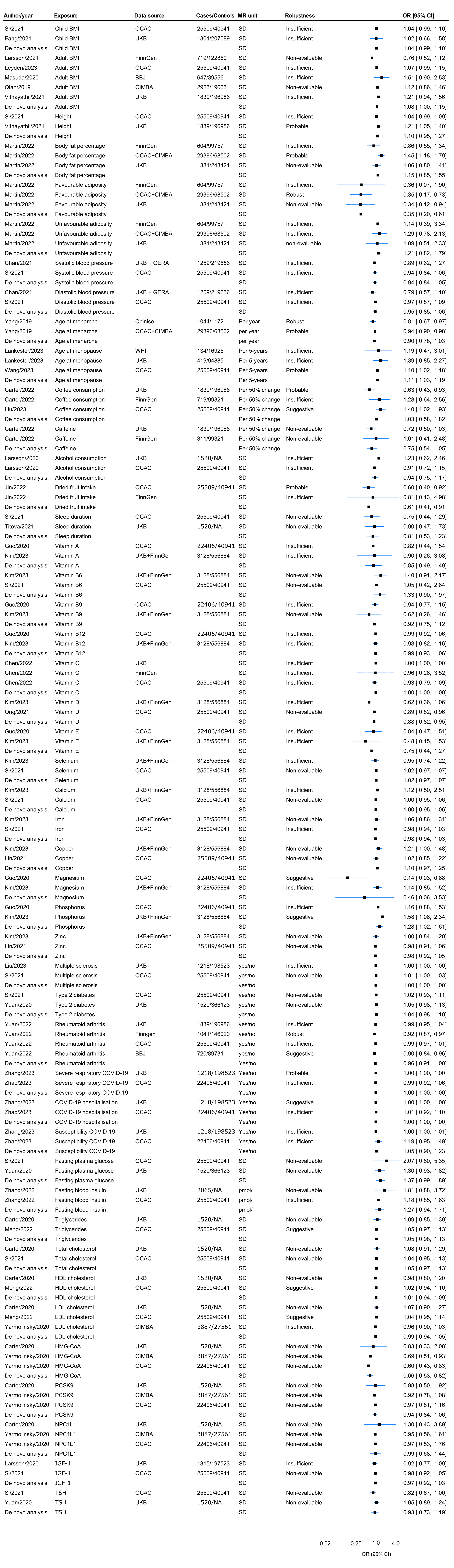
**Panel A.

Panel B.


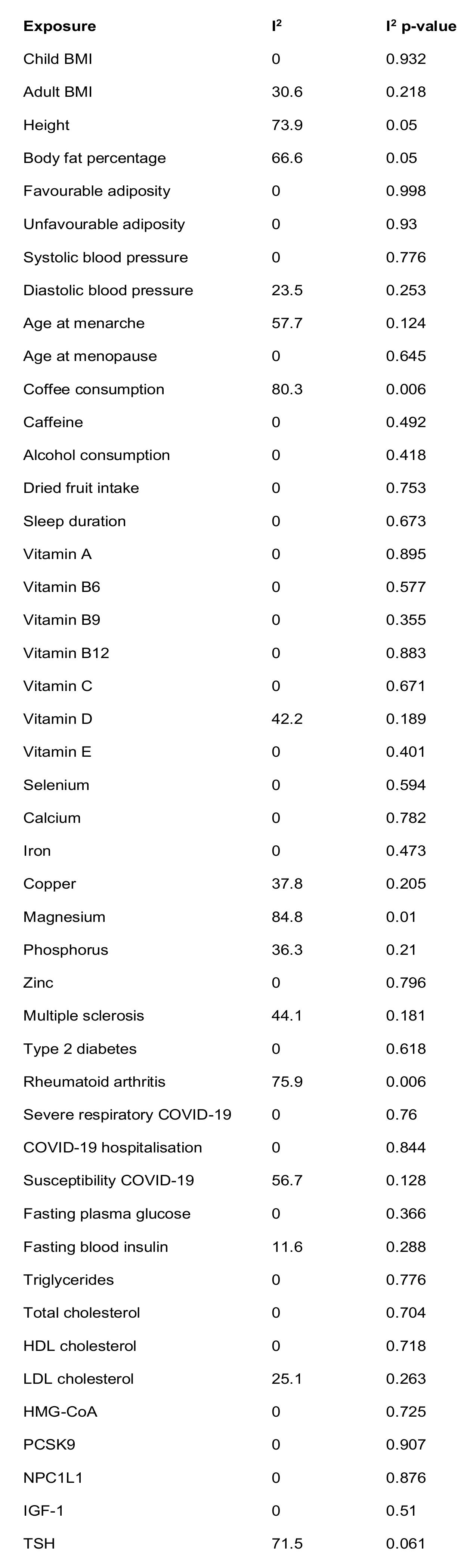


**Supplementary Figure 2**: All exposures included in de novo analysis: **Panel A**. The effect estimates with contributing cohorts. **Pannel B**. The heterogeneity statistics (I^2^ and I^2^ p-value). Note: For BMI de novo analysis, the lower bound of 95% confidence interval was 1.002, and is displayed as 1.00 due to rounding.

**References**

1. Moher D, Shamseer L, Clarke M, Ghersi D, Liberati A, Petticrew M, Shekelle P, Stewart LA. Preferred reporting items for systematic review and meta-analysis protocols (PRISMA-P) 2015 statement. *Systematic reviews* 2015;**4**: 1-9.

2. Shamseer L, Moher D, Clarke M, Ghersi D, Liberati A, Petticrew M, Shekelle P, Stewart LA. Preferred reporting items for systematic review and meta-analysis protocols (PRISMA-P) 2015: elaboration and explanation. *Bmj* 2015;**349**.

3. Babineau J. Product review: Covidence (systematic review software). *Journal of the Canadian Health Libraries Association/Journal de l'Association des bibliothèques de la santé du Canada* 2014;**35**: 68-71.

4. Markozannes G, Kanellopoulou A, Dimopoulou O, Kosmidis D, Zhang X, Wang L, Theodoratou E, Gill D, Burgess S, Tsilidis KK. Systematic review of Mendelian randomization studies on risk of cancer. *BMC medicine* 2022;**20**: 41.

5. Si S, Li J, Tewara MA, Li H, Liu X, Li Y, Chen X, Liu C, Yuan T, Li W, et al. Identifying causality, genetic correlation, priority and pathways of large-scale complex exposures of breast and ovarian cancers. *British journal of cancer* 2021;**125**: 1570‐81.

6. Mariosa D, Smith-Byrne K, Richardson TG, Ferrari P, Gunter MJ, Papadimitriou N, Murphy N, Christakoudi S, Tsilidis KK, Riboli E, Muller D, Purdue MP, et al. Body Size at Different Ages and Risk of 6 Cancers: A Mendelian Randomization and Prospective Cohort Study. *Journal of the National Cancer Institute* 2022;**114**: 1296-300.

7. Martin S, Tyrrell J, Thomas EL, Bown MJ, Wood AR, Beaumont RN, Tsoi LC, Stuart PE, Elder JT, Law P, Houlston R, Kabrhel C, et al. Disease consequences of higher adiposity uncoupled from its adverse metabolic effects using Mendelian randomisation. *eLife* 2022;**11**: e72452.

8. Freuer D, Linseisen J, O'Mara TA, Leitzmann M, Baurecht H, Baumeister S-E, Meisinger C. Body Fat Distribution and Risk of Breast, Endometrial, and Ovarian Cancer: A Two-Sample Mendelian Randomization Study. *Cancers* 2021;**13**: 5053.

9. Yarmolinsky J, Relton CL, Lophatananon A, Muir K, Menon U, Gentry-Maharaj A, Walther A, Zheng J, Fasching P, Zheng W, Yin Ling W, Park SK, et al. Appraising the role of previously reported risk factors in epithelial ovarian cancer risk: A Mendelian randomization analysis. *PLoS medicine* 2019;**16**: 1-22.

10. Ji Y, Yiorkas AM, Frau F, Mook-Kanamori D, Staiger H, Thomas EL, Atabaki-Pasdar N, Campbell A, Tyrrell J, Jones SE, Beaumont RN, Wood AR, et al. Genome-Wide and Abdominal MRI Data Provide Evidence That a Genetically Determined Favorable Adiposity Phenotype Is Characterized by Lower Ectopic Liver Fat and Lower Risk of Type 2 Diabetes, Heart Disease, and Hypertension. *Diabetes* 2019;**68**: 207-19.

11. Martin S, Cule M, Basty N, Tyrrell J, Beaumont RN, Wood AR, Frayling TM, Sorokin E, Whitcher B, Liu Y, Bell JD, Thomas EL, et al. Genetic Evidence for Different Adiposity Phenotypes and Their Opposing Influences on Ectopic Fat and Risk of Cardiometabolic Disease. *Diabetes* 2021;**70**: 1843-56.

12. Vithayathil M, Carter P, Kar S, Mason AM, Burgess S, Larsson SC. Body size and composition and risk of site-specific cancers in the UK Biobank and large international consortia: A mendelian randomisation study. *PLoS Medicine* 2021;**18**: 1-20.

13. Yang H, Dai H, Li L, Wang X, Wang P, Song F, Zhang B, Chen K. Age at menarche and epithelial ovarian cancer risk: A meta-analysis and Mendelian randomization study. *Cancer Medicine* 2019;**8**: 4012-22.

14. Wang Z, Lu J, Weng W, Zhang J. Women's reproductive traits and cerebral small-vessel disease: A two-sample Mendelian randomization study. *Frontiers in Neurology* 2023;**14**: 1064081.

15. Yarmolinsky J, Relton CL, Lophatananon A, Muir K, Menon U, Gentry-Maharaj A, Walther A, Zheng J, Fasching P, Zheng W, Ling WY, Park SK, et al. Appraising the role of previously reported risk factors in epithelial ovarian cancer risk: A Mendelian randomization analysis. *PLoS Medicine* 2019;**16**: e1002893.

16. Fan Z, Song H, Yuan R, Peng Y, Jiang Y. Genetic predisposition to female infertility in relation to epithelial ovarian and endometrial cancers. *Postgraduate medical journal* 2023;**99**: 63-8.

17. Yuan S, Chen J, Ruan X, Vithayathil M, Kar S, Li X, Mason AM, Burgess S, Larsson SC. Rheumatoid arthritis and risk of site-specific cancers: Mendelian randomization study in European and East Asian populations. *Arthritis Research and Therapy* 2022;**24**: 270.

18. Adams CD, Neuhausen SL. Bi-directional Mendelian randomization of epithelial ovarian cancer and schizophrenia and uni-directional Mendelian randomization of schizophrenia on circulating 1- or 2-glycerophosphocholine metabolites. *Molecular Genetics and Metabolism Reports* 2019;**21**.

19. Wen Y, Wu X, Peng H, Li C, Jiang Y, Liang H, Zhong R, Liu J, He J, Liang W. Cancer risks in patients with vitiligo: a Mendelian randomization study. *Journal of Cancer Research and Clinical Oncology* 2020;**146**: 1933-40.

20. Went M, Sud A, Mills C, Hyde A, Culliford R, Law P, Vijayakrishnan J, Gockel I, Maj C, Schumacher J, Palles C, Kaiser M, et al. Risk factors for eight common cancers revealed from a phenome-wide Mendelian randomisation analysis of 378,142 cases and 485,715 controls. *medRxiv* 2023.

21. Rueda-Martinez A, Garitazelaia A, Cilleros-Portet A, Mari S, Arauzo R, de Miguel J, Gonzalez-Garcia BP, Fernandez-Jimenez N, Bilbao JR, Garcia-Santisteban I. Genetic contribution of endometriosis to the risk of developing hormone-related cancers. *International journal of molecular sciences* 2021;**22**.

22. Mortlock S, Corona RI, Kho PF, Pharoah P, Seo JH, Freedman ML, Gayther SA, Siedhoff MT, Rogers PAW, Leuchter R, Walsh CS, Cass I, et al. A multi-level investigation of the genetic relationship between endometriosis and ovarian cancer histotypes. *Cell Reports Medicine* 2022;**3**: 100542.

23. Harris HR, Cushing-Haugen KL, Webb PM, Nagle CM, Jordan SJ, Group AOCS, Risch HA, Rossing MA, Doherty JA, Goodman MT, Modugno F, Ness RB, et al. Association between genetically predicted polycystic ovary syndrome and ovarian cancer: a Mendelian randomization study. *International Journal of Epidemiology* 2019;**48**: 822-30.

24. Yuan K, Song W, Liu Z, Lin GN, Yu S. Mendelian Randomization and GWAS Meta Analysis Revealed the Risk-Increasing Effect of Schizophrenia on Cancers. *Biology* 2022;**11**.

25. Zhang K, Liu X, Fu P, Zhao Y, Yu Q, Liu S, Xue F. Phenome-wide association study to explore the long-term symptoms after infection with novel coronavirus in the UK Biobank. *medRxiv* 2023.

26. Liu S, Feng S, Du F, Zhang K, Shen Y. Association of smoking, alcohol, and coffee consumption with the risk of ovarian cancer and prognosis: a mendelian randomization study. *BMC Cancer* 2023;**23**: 1-10.

27. Zhou Y, Zhou X, Sun J, Wang L, Zhao J, Chen J, Yuan S, He Y, Timofeeva M, Spiliopoulou A, Mesa-Eguiagaray I, Farrington SM, et al. Exploring the cross-cancer effect of smoking and its fingerprints in blood DNA methylation on multiple cancers: A Mendelian randomization study. *International Journal of Cancer* 2023;**153**: 1477-86.

28. Carter P, Yuan S, Kar S, Vithayathil M, Mason AM, Burgess S, Larsson SC. Coffee consumption and cancer risk: a Mendelian randomisation study. *Clinical Nutrition* 2022;**41**: 2113-23.

29. Wang J, Zhao H, Zhu J, Jiang M. Causal effects of physical activity on the risk of overall ovarian cancer: A Mendelian randomization study. *Digital Health* 2023;**9**.

30. Wang J, Zhao H, Zhu J, Jiang M. Causal effects of physical activity on the risk of overall ovarian cancer: A Mendelian randomization study. *Digital health* 2023;**9**: 20552076231162988.

31. Chen J, Yang K, Qiu Y, Lai W, Qi S, Wang G, Chen L, Li K, Zhou D, Liu Q, Tang L, Liu X, et al. Genetic associations of leisure sedentary behaviors and the risk of 15 site-specific cancers: A Mendelian randomization study. *Cancer Medicine* 2023;**12**: 13623-36.

32. Guo Y, Lu Y, Jin H. Appraising the role of circulating concentrations of micro-nutrients in epithelial ovarian cancer risk: a Mendelian randomization analysis. *Scientific Reports* 2020;**10**: 7356.

33. Kim JY, Song M, Kim MS, Natarajan P, Do R, Myung W, Won H-H. An atlas of associations between 14 micronutrients and 22 cancer outcomes: Mendelian randomization analyses. *BMC Medicine* 2023;**21**: 1-12.

34. Johansson Å, Schmitz D, Höglund J, Hadizadeh F, Karlsson T, Ek WE. Investigating the Effect of Estradiol Levels on the Risk of Breast, Endometrial, and Ovarian Cancer. *Journal of the Endocrine Society* 2022;**6**: 1-9.

35. Meng H, Wang R, Song Z, Wang F. Causal Effects of Circulating Lipid Traits on Epithelial Ovarian Cancer: A Two-Sample Mendelian Randomization Study. *Metabolites* 2022;**12**: 1175.

36. Yarmolinsky J, Bull CJ, Vincent EE, Robinson J, Walther A, Smith GD, Lewis SJ, Relton CL, Martin RM. Association Between Genetically Proxied Inhibition of HMG-CoA Reductase and Epithelial Ovarian Cancer. *JAMA: Journal of the American Medical Association* 2020;**323**: 646-55.

37. Long Y, Tang L, Zhou Y, Zhao S, Zhu H. Causal relationship between gut microbiota and cancers: a two-sample Mendelian randomisation study. *BMC Medicine* 2023;**21**: 66.

38. Jayarathna DK, Renteria ME, Malik A, Sauret E, Batra J, Gandhi NS. Integrative Transcriptome-Wide Analyses Uncover Novel Risk-Associated MicroRNAs in Hormone-Dependent Cancers. *Frontiers in Genetics* 2021;**12**.

39. Yarmolinsky J, Robinson JW, Mariosa D, Karhunen V, Huang J, Dimou N, Murphy N, Burrows K, Bouras E, Smith-Byrne K, Lewis SJ, Galesloot TE, et al. Association between circulating inflammatory markers and adult cancer risk: a Mendelian randomization analysis. *medRxiv* 2023.

40. Feng Y, Wang R, Li C, Cai X, Huo Z, Liu Z, Ge F, Huang C, Lu Y, Zhong R, Li J, Cheng B, et al. Causal effects of genetically determined metabolites on cancers included lung, breast, ovarian cancer, and glioma: a Mendelian randomization study. *Translational Lung Cancer Research* 2022;**11**: 1302-14.

41. Bouras E, Karhunen V, Gill D, Huang J, Haycock PC, Gunter MJ, Johansson M, Brennan P, Key T, Lewis SJ, Martin RM, Murphy N, et al. Circulating inflammatory cytokines and risk of five cancers: a Mendelian randomization analysis. *BMC Medicine* 2022;**20**: 3-.

42. Considine DPC, Jia G, Shu X, Schildkraut JM, Pharoah PDP, Zheng W, Kar SP, Ovarian Cancer Association C. Genetically predicted circulating protein biomarkers and ovarian cancer risk. *Gynecologic oncology* 2021;**160**: 506-13.
